# Single-Channel Electrophysiology Reveals a Distinct and Uniform Pore Complex Formed by α-Synuclein Oligomers in Lipid Membranes

**DOI:** 10.1371/journal.pone.0042545

**Published:** 2012-08-03

**Authors:** Felix Schmidt, Johannes Levin, Frits Kamp, Hans Kretzschmar, Armin Giese, Kai Bötzel

**Affiliations:** 1 Neurologische Klinik, Klinikum der Universität München, Ludwig-Maximilians-Universität München, München, Germany; 2 Zentrum für Neuropathologie und Prionforschung, Ludwig-Maximilians-Universität München, München, Germany; 3 Deutsches Zentrum für Neurodegenerative Erkrankungen und Adolf-Butenandt-Institut, Ludwig-Maximilians-Universität München, München, Germany; Georgia Health Sciences University, United States of America

## Abstract

Synucleinopathies such as Parkinson's disease, multiple system atrophy and dementia with Lewy bodies are characterized by deposition of aggregated α-synuclein. Recent findings indicate that pathological oligomers rather than fibrillar aggregates may represent the main toxic protein species. It has been shown that α-synuclein oligomers can increase the conductance of lipid bilayers and, in cell-culture, lead to calcium dyshomeostasis and cell death. In this study, employing a setup for single-channel electrophysiology, we found that addition of iron-induced α-synuclein oligomers resulted in quantized and stepwise increases in bilayer conductance indicating insertion of distinct transmembrane pores. These pores switched between open and closed states depending on clamped voltage revealing a single-pore conductance comparable to that of bacterial porins. Pore conductance was dependent on transmembrane potential and the available cation. The pores stably inserted into the bilayer and could not be removed by buffer exchange. Pore formation could be inhibited by co-incubation with the aggregation inhibitor baicalein. Our findings indicate that iron-induced α-synuclein oligomers can form a uniform and distinct pore species with characteristic electrophysiological properties. Pore formation could be a critical event in the pathogenesis of synucleinopathies and provide a novel structural target for disease-modifying therapy.

## Introduction

Synucleinopathies, such as multiple system atrophy (MSA), dementia with Lewy bodies (DLB) and Parkinson's disease (PD) are characterized by deposits of aggregated α-synuclein (α-syn) in the brain [1], [2]. Mutations and amplifications of the α-syn gene in familial PD as well as GWAS studies in sporadic PD indicate that α-syn plays a key role in disease pathogenesis [3]. Although the underlying mechanism of cell death remains unclear, accumulating evidence indicates that disease specific α-syn aggregate formation is a critical event. Pathological oligomers that are formed on pathway to fibrillar aggregates seem to be the most important toxic species [4]–[6]. Notably, overexpression of human α-syn is sufficient to cause apoptosis and damage of cell organelles, without detectable fibril formation [7], [8].

In PD patients, an increase of iron levels has been found in brain regions affected by neurodegeneration [9]. Fe^3+^ seems to play a pivotal role in α-syn aggregation since it is able to trigger the formation of distinct oligomers of α-syn *in vitro* [10]. Correspondingly, in cell culture, Fe^3+^ was able to induce oligomerization of α-syn leading to cytotoxicity [11]. Moreover, it was shown that iron-induced α-syn oligomers enhance pre- and postsynaptic transmission, alter intracellular calcium homeostasis and lead to cell death in primary cultures of cortical neurons [12]. Interestingly, the formation of this oligomer species is favored by oxidative stress [13]

One possible mechanism of oligomer toxicity is the formation of lipid bilayer permeabilizing pores [14] resulting in cytotoxicity [10]–[12]. Indeed, oligomers dissociated from preformed α-syn fibrils were able to increase the conductance of lipid bilayers [15]. Formation of pores by oligomeric intermediates may be a fundamental mechanism of cell death in a large range of neurodegenerative diseases. Morphologically, the formation of annular pore-like structures was shown for several proteins deposited in various neurodegenerative diseases including α-syn, Aβ, prion protein and polyglutamine-containing proteins. Moreover, in some instances an increase in the permeability of membranes could be shown [14].

An important question remaining to be addressed is whether the binding of pathological protein aggregates to membranes results in an unspecific “diffuse” membrane damage, like membrane thinning or disruption [16], [17], or in the formation of distinct pores. The aim of this study was to characterize the potential pores formed by α-syn oligomers at the single-channel level to resolve this issue. The finding of a distinct and surprisingly uniform pore species might provide a promising novel structural target for therapeutic intervention in neurodegenerative diseases.

## Materials and Methods

### Expression and purification of recombinant human α-syn

Expression and purification was performed as described previously [10], [18], using pET-5a/α-synuclein (136TAT) plasmid (wt-plasmid by Philipp Kahle, LMU Munich; the 136-TAC/TAT-Mutation was performed by Matthias Habeck, ZNP Munich). The purified protein was adjusted to 1 mg/ml by dilution in 50 mM Tris-HCl, pH = 7.0 and stored at −80°C after freezing in liquid nitrogen.

### Preparation of α-syn oligomers

Recombinant human α-syn was incubated in 50 mM Tris-HCl, pH = 7.0 with 1% DMSO (Sigma-Aldrich, Taufkirchen, Germany) and 20 µM FeCl_3_ (Merck, Darmstadt, Germany) without agitation at RT in a total volume of 200 µl using various concentrations and incubation times, with or without inhibitors of protein aggregation.

### Inhibition of pore formation

Baicalein was previously shown to strongly inhibit aggregation of α-syn *in vitro* and α-syn toxicity in cell culture [10], [19]. Therefore we tested its influence on pore formation by co-incubation of α-syn with 50 µM baicalein in presence of 1% DMSO (Sigma) and 20 µM FeCl_3_ (Merck).

### Influence of α-syn on electrophysiological properties of planar lipid bilayers

Planar lipid bilayers were produced in the Ionovation Compact (Ionovation, Osnabrück, Germany) by the painting technique [20]. Two bath chambers separated by a Teflon-septum were filled with 250 mM KCl, 10 mM MOPS, pH = 7.2 (Merck). In the *cis-*chamber, 2 µl of a 100 mg/ml-solution of purified azolectin in n-Decane (Ionovation) was applied to a pinhole of 120 µm in diameter. After 30 min incubation at RT, lipid was thinned out by repetitive lowering and re-raising of the buffer-level until a bilayer was formed. Bilayer formation was monitored optically and by capacitance- and conductance-measurements. The resulting bilayers had a typical capacitance of 60–80pF and a resistance of >100GΩ. The monitoring of the bilayer was performed using Ag/AgCl-electrodes (Ionovation), an EPC 10-amplifier and Patchmaster-software (HEKA, Lambrecht/Pfalz, Germany). The electrode in the *cis*-chamber was directly connected to the amplifier, so all potentials are referred to this compartment. The noise was ∼0.4pA (r.m.s.) at 3 kHz bandwidth.

**Figure 1:**
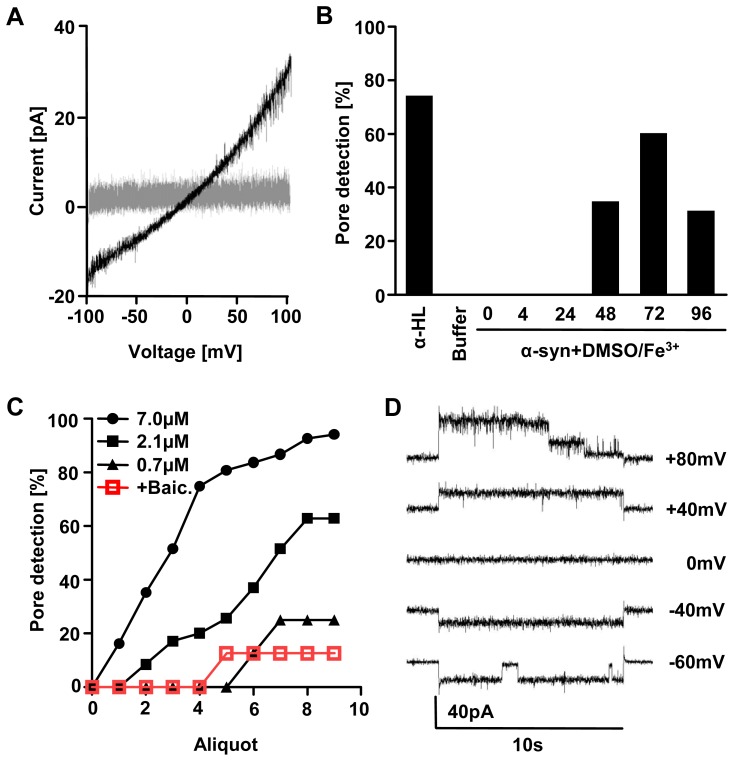
Detection of α-syn pores. **A**) Pore formation results in increased current-flows over the membrane (black trace) compared to intact bilayers (grey trace) when a voltage ramp is applied. **B**) Pore detection rate for oligomers obtained with 2.1 µM α-syn with 1% DMSO and 20 µM Fe^3+^ at RT. 4 and 24 h of incubation did not lead to any pore detections (N = 8, each). In 33% of all 48 h-samples (N = 9) pore formations could be detected, increasing to a maximum at 72 h (N = 10). Further incubation results in a decrease of pore detection (N = 10). Non-incubated α-syn monomers did not lead to pore detections (N = 4), as well as DMSO and Fe^3+^ (“buffer”) after all tested incubation times (N = 4, each). α-hemolysin was used as a positive control (α-HL; N = 4). **C**) In a further set of experiments, the effects of different α-syn concentrations and the effect of the aggregation inhibitor baicalein were investigated after incubation of α-syn for 72 h with DMSO and Fe^3+^. Shown is the probability of pore detection following sequential application of aliquots of the respective samples to the bilayer. α-syn was used at 7.0 µM (N = 69), 2.1 µM (N = 35) and 0.7 µM (N = 8) with up to 9 aliquots applied per sample. With decreasing α-syn concentration, cumulative pore detection rate decreases significantly (p<0.001). Co-incubation of 2.1 µM of α-syn with 50 µM of baicalein (N = 8) significantly decreases pore detection compared to 2.1 µM control condition (p<0.005). **D**) When different voltages were clamped to membranes with inserted pores, step-like changes in conductivity were consistently observed during the duration of the voltage-pulse.

After bilayer formation, we waited for 10 min to ensure application of the protein to a stable bilayer-system. Then, α-syn aggregation samples (total assay volume: 200 µl) were added in aliquots of 20 µl close to the membrane in the *trans*-chamber. The electrophysiological properties were monitored using +/−20 mV-squarewave-voltage pulses. Pore formation resulted in an increase in the current flow over the membrane compared to an intact bilayer (Fig. 1A). Threshold for pore detection was set to a conductance of 70pS. If no increase in bilayer conductance beyond the threshold was detected for 5 min, the next aliquot of the sample was added. Pore detection rate was defined as the probability of pore detection per α-syn aggregation sample. In the event of an increase in bilayer conductance, a standardized recording-protocol was employed, consisting of a voltage-ramp reaching from −100 to +100 mV over 10 sec and different squarewave-voltage pulses (10 sec each at −60/−50/−40/−30/−20/−10/0/+10/+20/+30/+40/+50/+60 mV, 5× −40 mV, 5× +40 mV, −60/−70/−80/−90/−100/−110/−120 mV, 5× −80 mV, +60/+70/+80/+90/+100/+110/+120 mV, 5× +80 mV)) to characterize the potential α-syn pore electrophysiologically. Conductance was calculated from the voltage-current-recordings and corrected for the current noise at 0 mV using a Matlab-based program (The MathWorks, Natick, MA, USA).

### Influence of different cations on pore-conductance

Following pore characterization in presence of KCl (see above), in some experiments the chamber-buffer was perfused with 30 ml of 250 mM NaCl, 10 mM MOPS, pH = 7.2 (Merck) with 7.2 ml/min in both chambers until the buffer was completely exchanged to NaCl buffer. Then the same squarewave-voltage pulse protocol as in presence of KCl was used.

### Statistical analysis

Statistical analysis was carried out using SigmaStat 3.5 software (Systat Software, Erkrath, Germany). Threshold for significance was set to p<0.05.

## Results

### Time-course and concentration-dependency of pore formation and detection

In this study, changes in the permeability of a planar lipid membrane upon addition of preformed oligomers of α-syn were monitored. First we optimized aggregation conditions towards maximum pore detection rate. For incubation times of 4 h and 24 h no pore formation was observed. However, pore detection rate increased to a maximum after 72 h of incubation. Further incubation (96 h) resulted in a decrease of the pore detection rate (Fig. 1B). Non-incubated monomeric α-syn did not lead to pore detections, as well as DMSO and Fe^3+^ in the absence of α-syn at all tested incubation times. α-hemolysin was used as a positive control for the formation of distinct oligomer pores in lipid-bilayers. Additionally, pore detection was dependent of protein concentration. 7 µM of α-syn incubated resulted in a cumulative pore detection rate of >90% over all applied aliquots of the aggregation sample, decreasing for concentrations of 2.1 and 0.7 µM (Fig. 1C).

### Inhibiton of pore formation

As pore formation by α-syn seemed to be in the dynamic range for a protein concentration of 2.1 µM, we tested the influence of baicalein on pore formation at this concentration. Co-incubation of α-syn with baicalein resulted in a significantly decreased pore detection compared to control conditions (Fig. 1C).

### Conductance steps observed for α-syn pores

**Figure 2:**
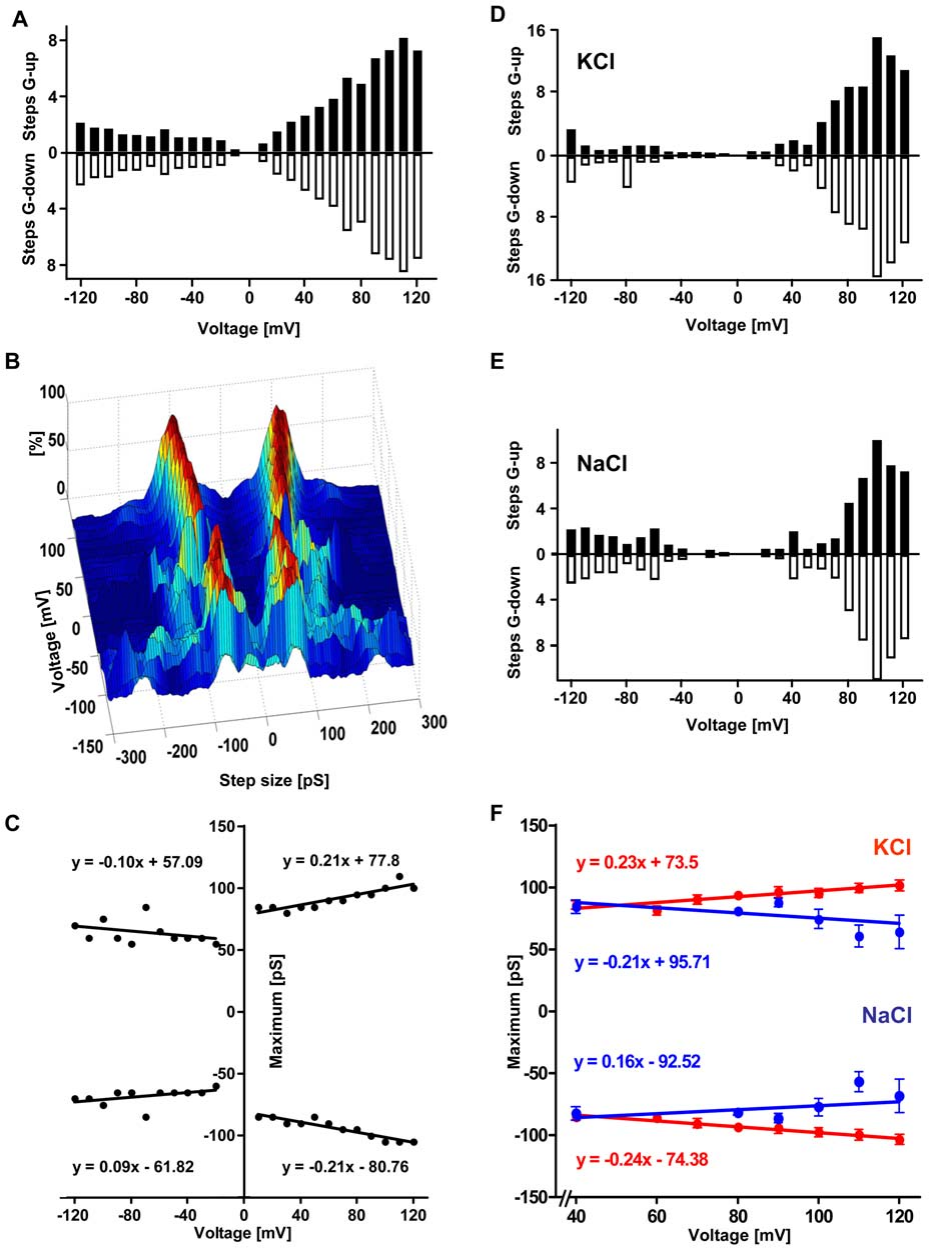
Characterization of α-syn pores. **A**) Depending on clamped voltage, the number of steps increasing or decreasing pore conductance (“G-up” and “G-down” refer to the number of steps per recorded trace of 10s) differs (N = 18804 steps in 74 pore formations with 42 traces each). At higher voltages, more steps can be detected per trace. For positive voltages, more steps are observed than for negative voltages. **B**) A histogram summarizing the distribution of all measured conductance-steps (N = 18804) reveals a symmetrical distribution of steps with a well-defined peak for the main step-size of +/−50 to +/−100pS depending on the clamped voltage (as the number of steps was different for the different voltages, histograms were normalized by setting maxima to 100%). **C**) Maxima of step size distribution are plotted against clamped voltage. For positive voltages, step size is higher than for negative clamped voltages and increases linear with clamped voltage with a significant positive correlation (p≤0.0001). **D–E**) All pores characterized both in KCl- and NaCl-buffer show a transmembrane current flow after buffer exchange (N = 9). After exchange to NaCl-buffer, conductance steps are still observed with a similar rate and distribution as in KCl-buffer (see also Fig. 1A). **F**) For positive clamped voltages, the conductance increases linear with the clamped voltage in KCl-buffer (see also Fig. 1C, p≤0.0001). In contrast, lower conductance levels for high positive voltages are observed in NaCl-buffer that decrease linear with clamped voltage (p≤0.0005).

Having established optimal pre-incubation conditions to generate pore forming α-syn oligomers, we performed all further experiments using 7 µM protein concentration and 72 h of incubation at RT. By applying voltage pulses to membranes with an inserted pore, and analyzing current flows with a standardized protocol (see Materials and Methods) we consistently observed distinct step-like changes of conductivity during the duration of the voltage pulse (Fig. 1D). For 74 pore formation events, by plotting the number of all steps per recorded trace against the clamped voltage, an asymmetric behavior of the pore conductance G became apparent (Fig. 2A). First higher voltages resulted in more steps per trace (up to approx. 1step/sec). Second for positive clamped voltages considerably more steps were observed than for negative voltages. Third there was a slight shift to more G-decreasing events at high positive voltages.

As conductance-steps were voltage-dependent, histograms of the distribution of step sizes were calculated separately for different voltages. The distribution of step-sizes stratified for voltage from 74 independent experiments available for detailed analysis are summarized in Fig. 2B which shows two distinct main peaks distributed symmetrically around 0pS. A voltage-dependency of pore-conductance is evident, when the maxima of the distribution of step sizes are plotted against the clamped voltage (Fig. 2C). For positive voltages the step-size is higher and shows a significant positive correlation with the clamped voltage.

### Cation-dependency of pore-conductance

We found that conductance was still increased after complete exchange of the chamber-buffer from KCl to NaCl in all pore detections analyzed under both conditions. Moreover, conductance steps still arise in a similar voltage-dependent distribution (Fig. 2D, E). However, K^+^→Na^+^ exchange led to a change in pore-conductance. Plotting the maximum of the distribution of conductance steps (see also Fig. 2B, C) in presence of NaCl or KCl, conductance was increased at higher positive voltages for KCl, but decreased for NaCl (Fig. 2F).

### Detailed analysis of conductance steps at +80 Mv

**Figure 3:**
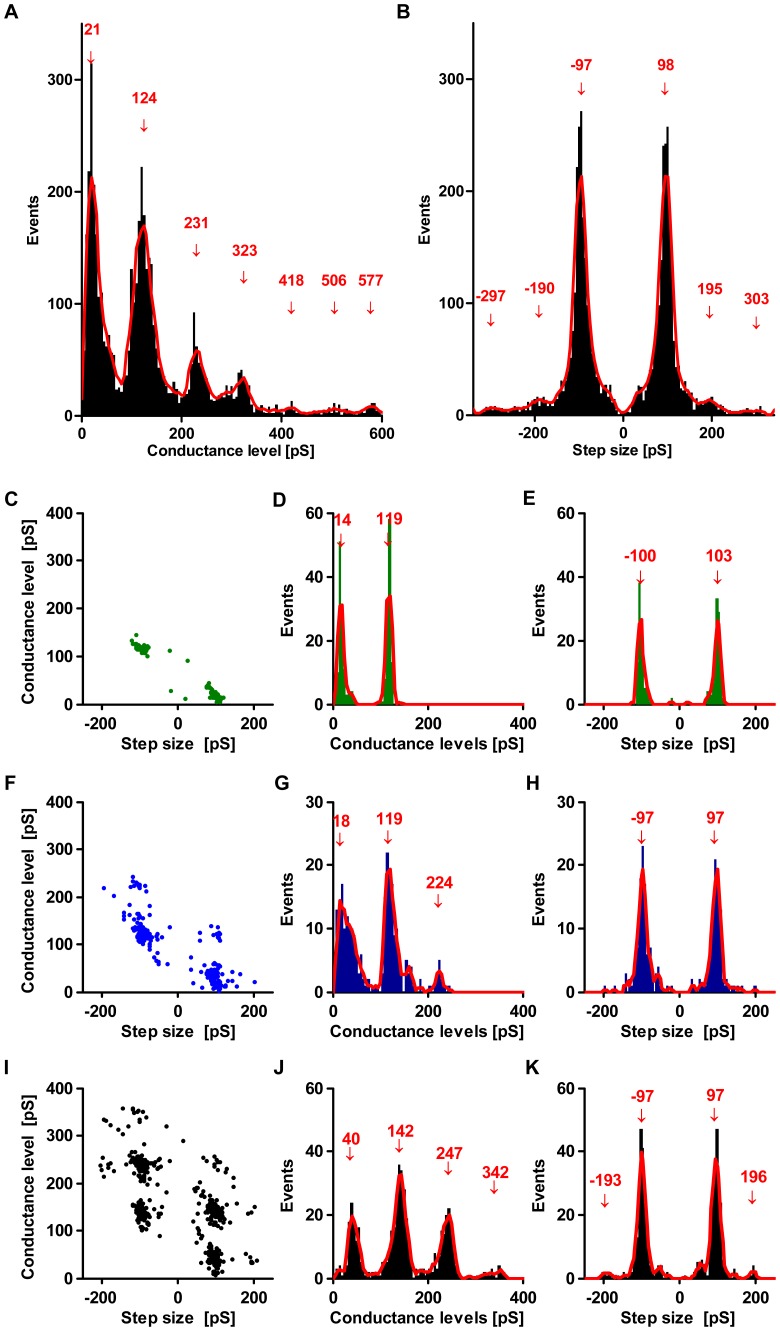
Distribution of conductance levels and conductance steps measured at +80 mV. **A**) Histogram analysis of all conductance levels measured at +80 mV for all experiments with pore detection (N = 4435 steps in 74 pore formations) shows a quantized distribution of conductance levels with a quantum unit of ∼100pS. The red line represents the running-mean over 8 values, arrows indicate maxima. **B**) The distribution of corresponding step sizes found in these measurements indicates a common quantized step size of ∼100pS and multiples of this. Panels **C–K** show the data of individual experiments with putative insertion of either one (**C–E**), two (**F–H**) or three pores (**I–K**). The leftmost panel (**C, F, I**) shows that conductance levels and corresponding consecutive steps in conductance are not independent from each other, but show a clear clustering of data points. The histograms of conductance levels in the middle panel (**D, G, J**) show different numbers of distinct peaks depending on the number of inserted pores but not differing in quantization with a common unit of ∼100pS. The histograms of observed conductance steps (**E, H, K**) show a common main step size of ∼100pS and, rarely, multiples of this unit in experiments with more than one pore inserted into the membrane.

The highest number of conductance steps (N = 4435) was recorded at +80 mV due to voltage-dependency and a higher number of traces recorded at this voltage. Thus, detailed quantitative analysis of the distribution of conductance steps and conductance levels was performed for this voltage (Fig. 3A, B). The histogram of conductance levels shows distinct peaks that differ from each other by ∼100pS, indicating quantization of conductivity with a smallest step of ∼100pS. Correspondingly, the distribution of step sizes shows a well-defined maximum at ∼100pS with very small additional maxima at multiples of this quantum unit. These quantized steps and conductance levels can be explained by changes in the number of open pores with similar conductance inserted in the bilayer. To further scrutinize this model, we used the data obtained in individual experiments and analyzed the correlation between number and magnitude of conductance levels and step sizes. Figure 3C–K shows examples from 3 representative individual experiments differing in regard to the number of distinct conductance levels observed. In these experiments, either two (Fig. 3C–E), three (Fig. 3F–H) or four (Fig. 3I–K) peaks in the distribution of conductance levels were seen. However, independent of the number of maxima of conductance levels, these maxima always differed by a step size of ∼100pS. This fits well with a model based on changes in the number of open pores being present, whereas the inter-experimental variation in the number of conductance levels observed would be difficult to accommodate in a model based on different conductance level of one individual pore. Thus, these results could best be explained by the presence of either 0–1, 0–2 or 0–3 open pores in the experiments shown in Figure 3C–E, F–H and I–K, respectively. Notably, the conductance level for no open pore inserted is slightly above zero and highest in the experiment with up to three open pores being detected.

## Discussion

Pore formation has been proposed as a key mechanism of oligomer toxicity in neurodegenerative diseases which are characterized by pathological protein aggregation. At the morphological level, formation of quite uniform annular oligomers has been described for several amyloidogenic proteins [14]. However, a detailed electrophysiological characterization at the single-channel level has been missing.

We previously provided a detailed morphological and structural characterization of iron-induced α-syn oligomers based on single-particle spectroscopy and atomic force microscopy [10]. It was shown that these oligomers are able to interact with lipid membranes, to permeabilize lipid vesicles and to increase the conductance of lipid bilayers [10], [21], [22]. Notably, membrane permeabilization could be blocked with the oligomer-specific antibody A11 [4], [10]. Moreover, we showed that iron-induced oligomers modulate electrophysiological properties of neuronal cells and are toxic in cell culture [11], [12]. Beyond this, baicalein, an inhibitor of α-syn aggregation [19] that we previously showed to inhibit the formation of iron-induced membrane-binding α-syn oligomers and toxicity in cell culture [10], [21], strongly inhibited pore formation in our electrophysiological study. In addition, we demonstrated that anle138b, a compound that specifically inhibits α-syn oligomer formation both *in vitro* and *in vivo* also inhibits α-syn induced membrane permeabilization in our lipid bilayer system and rescues the motor phenotype in animal models of PD [23]. Thus, we used this type of structurally and functionally well-characterized oligomers for detailed analysis of potential pore formation by single-channel electrophysiology.

**Figure 4:**
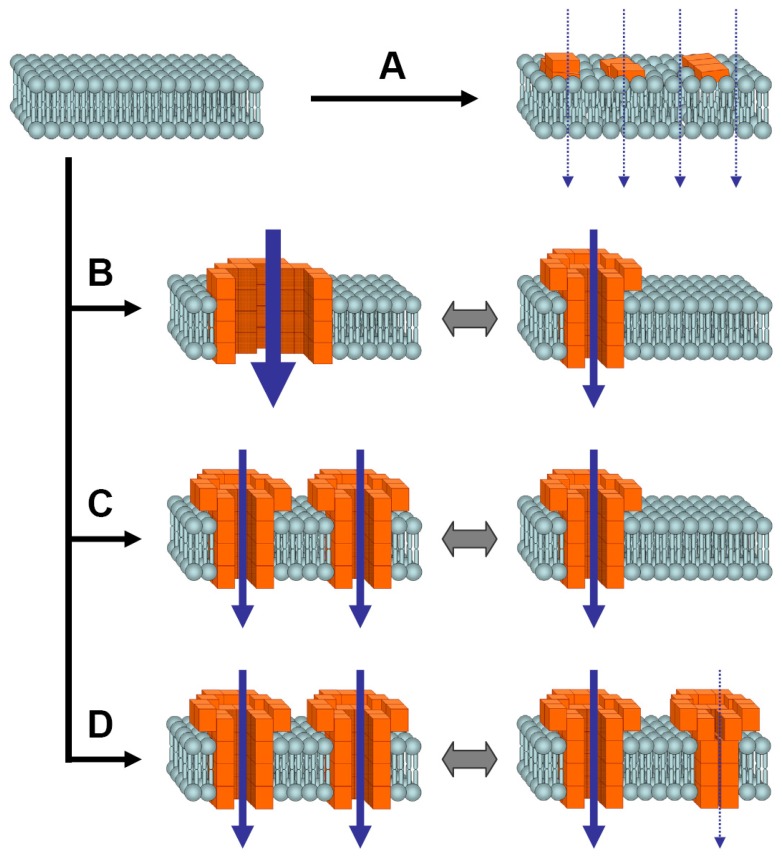
Schematic illustration of different models for increased membrane permeability caused by α-syn oligomers. In principle, α-syn oligomers could cause increased conductance of lipid membranes by different modes of action. **A**) A diffuse damage of the bilayer could lead to an unspecific increase in transmembrane current flow. **B**) Distinct pores could be formed in the bilayer that switch between two or more different conformational states, resulting in corresponding changes in conductivity. **C**) Different numbers of uniform pores could spontaneously insert and de-insert into the membrane leading to step-like changes in conductivity. **D**) The number of “open” pores could fluctuate due to open and closure events of permanently inserted pore complexes.

In principle, α-syn oligomers could affect membrane conductance by different molecular mechanisms (Fig. 4). It has been proposed that oligomers could increase lipid bilayer conductance by a “diffuse” damage to the bilayer (model A, Fig. 4A). However, we reproducibly observed discrete step-like changes in the transmembrane current traces (Fig. 1) and well-defined peaks in the distribution of conductance levels and step sizes (Fig. 2). These results would be difficult to explain by a model based on unspecific “diffuse” bilayer damage. In contrast, in a model based on distinct transmembrane oligomer pores, distinct conductance levels could easily be accommodated. Steps in conductance could be explained by different processes. First, a rapid conformational switch of individual transmembrane pores could result in multiple different conductance states (model B, Fig. 4B). Second, conductance-steps could be explained by fluctuations in the number of pores. These fluctuations could be either due to spontaneous insertion and de-insertion into the membrane (model C, Fig. 4C) or due to *open* and *closure* events of permanently inserted pores (model D, Fig. 4D). Model C can be excluded as conductance steps in both directions are preserved after complete buffer-exchange (Fig. 4D–F), which removes all non-inserted oligomers available in the chamber buffer for potential (re-)insertion into the membrane.

We found that the distribution of conductance-levels that differ from each other by a defined quantized step size (Fig. 3A, B). This suggests insertion of multiple uniform pores (model D) rather than many different conformational states of a single pore (model B). This reasoning is supported further by the detailed analysis of individual experiments. If multiple conductance states were caused by multiple states of one single pore, these states should, in principle, be consistently detectable in all individual experiments. However, we found that experiments differed in regard to the maximum conductance observed, but consistently showed the same quantization and the same step size (Fig. 3C–K), which argues against model B. As described for Fig. 3, our results can best be explained by stable insertion of variable numbers of uniform pores that switch between an *open* and a *closed* state in the individual experiments (model D). Opening and closure of pores may occur either independently or - more rarely – at the same time resulting in steps of ∼100pS (in the case of +80 mV clamped voltage) or multiples of this quantum unit. Of note, the baseline conductance of bilayers with all inserted pores being in the closed state (i.e. the leftmost histogram peak in Fig. 3D, G, J) seems to depend on the number of inserted pores. This finding suggests that also “closed” pores may result in a small leak current that slightly increases baseline conductance and provides a further piece of evidence arguing against model C.

Taken together, our results indicate that distinct and uniform pores are formed as a complex of α-syn molecules by a well defined assembly. Beyond this, these pore complexes share key properties with bacterial porins, which provide an example of oligomer pores optimized by evolution to mediate cellular toxicity. Interestingly, bacterial porins also show step-like changes of conductivity [24], [25] that have been interpreted as voltage-dependent open and closure events [25]–[27]. Due to its unidirectional incorporation into lipid bilayers, the conductance of Omp34-channels shows a dependency on the polarity of the clamped voltage [27]. Again, this is in line with our findings for α-syn pores, which further corroborates the concept of a distinct and uniform pore complex. The conductance of pores formed by α-syn oligomers in our study is only slightly less than conductances reported for bacterial pore-forming toxins (α-hemolysin 240pS [28], [29], Omp-porins 450pS [24] in comparable conditions), indicating that the resulting current flow could be sufficient to induce toxic effects. This reasoning is further supported by the finding that compounds that inhibited the formation of iron-induced α-syn oligomers and blocked oligomer-induced transmembrane currents *in vitro* also reduced toxicity in cell-culture- and *in vivo-*models of PD [10]–[12], [23].

## Conclusion

Taken together, our results indicate that α-syn oligomers can form a distinct and uniform pore complex. Notably, the α-syn pore complex identified by us shares several electrophysiological properties with bacterial porins such as the dependence of pore-conductance on both direction and magnitude of the clamped voltage and the available cation [27], [30], [31]. Because of its distinct and uniform electrophysiological properties, the α-syn pore complex described here might represent a specific particle species that could provide a novel target structure for the development of drugs that inhibit oligomer pore formation or modulate the electrophysiological properties of these pores.
